# *Dendrobium moniliforme* Exerts Inhibitory Effects on Both Receptor Activator of Nuclear Factor Kappa-B Ligand-Mediated Osteoclast Differentiation *in Vitro* and Lipopolysaccharide-Induced Bone Erosion *in Vivo*

**DOI:** 10.3390/molecules21030295

**Published:** 2016-03-01

**Authors:** Jong Min Baek, Ju-Young Kim, Sung-Jun Ahn, Yoon-Hee Cheon, Miyoung Yang, Jaemin Oh, Min Kyu Choi

**Affiliations:** 1 Department of Anatomy, School of Medicine, Wonkwang University, Iksan, Jeonbuk 570-749, Korea; phone8418@hanmail.net (J.M.B.); asj0427@naver.com (S.-J.A.); yangm@wku.ac.kr (M.Y.); jmoh@wku.ac.kr (J.O.); 2 Imaging Science-Based Lung and Bone Diseases Research Center, Wonkwang University, Iksan, Jeonbuk 570-749, Korea; Kimjy1014@gmail.com (J.-Y.K.); hanleuni@naver.com (Y.-H.C.); 3 Institute for Skeletal Disease, Wonkwang University, Iksan, Jeonbuk 570-749, Korea; 4 Institute for Environmental Science, Wonkwang University, Iksan, Jeonbuk 570-749, Korea

**Keywords:** *Dendrobium moniliforme* (DM), osteoclast, bone resorption, osteoporosis

## Abstract

*Dendrobium moniliforme* (DM) is a well-known plant-derived extract that is widely used in Oriental medicine. DM and its chemical constituents have been reported to have a variety of pharmacological effects, including anti-oxidative, anti-inflammatory, and anti-tumor activities; however, no reports discuss the beneficial effects of DM on bone diseases such as osteoporosis. Thus, we investigated the relationship between DM and osteoclasts, cells that function in bone resorption. We found that DM significantly reduced receptor activator of nuclear factor kappa-B ligand (RANKL)-induced tartrate-resistant acid phosphatase (TRAP)-positive osteoclast formation; DM directly induced the down-regulation of c-Fos and nuclear factor of activated T cells c1 (NFATc1) without affecting other RANKL-dependent transduction pathways. In the later stages of osteoclast maturation, DM negatively regulated the organization of filamentous actin (F-actin), resulting in impaired bone-resorbing activity by the mature osteoclasts. In addition, micro-computed tomography (μ-CT) analysis of the murine model revealed that DM had a beneficial effect on lipopolysaccharide (LPS)-mediated bone erosion. Histological analysis showed that DM attenuated the degradation of trabecular bone matrix and formation of TRAP-positive osteoclasts in bone tissues. These results suggest that DM is a potential candidate for the treatment of metabolic bone disorders such as osteoporosis.

## 1. Introduction

Osteoporosis is a severe metabolic disease associated with pathological changes in bone architecture, mineralization, strength, and composition. Since incidence rates of various symptoms of osteoporosis, such as fragility fractures and subsequent chronic pain, psychological depression, and physical impairments continue to escalate with the rapidly growing aging population, the development of novel therapeutic materials for the management of osteoporosis has been recognized as an important issue by the research community [1].

Several studies have discovered potential agents for treatment of osteoporosis by using natural, plant-derived products, because they have fewer side effects and higher efficacy in comparison with synthetic materials [2]. A variety of plant extracts, such as those of *Stauntonia hexaphylla*, *Angelica tenuissima*, and *Ampelopsis brevipedunculata*, have been demonstrated to have the potential to treat bone loss through the suppression of osteoclast differentiation and function; osteoclasts are bone-resorbing cells essential for bone metabolism [3,4,5]. However, there have been no reports on the effects of *Dendrobium moniliforme* (DM) extract on the activity of bone cells, particularly osteoclasts.

DM is the botanical name of *Dendrobium nobile* Lindl., in the family Orchidaceae. It has been widely used in traditional medicine for the treatment of various diseases [6,7]. Previous investigation demonstrated that DM exerts anti-oxidant effects *in vitro* and reno-protective effects in high fat diet-treated mice by reducing serum glucose levels, total cholesterol concentration, and renal lipid accumulation *in vivo* [8]. DM is composed of alkaloids that have anti-neuroinflammatory, anti-tumor, anti-platelet aggregation, and immunoregulatory effects [7,9,10].

In this study, in order to determine if DM has therapeutic effects against major metabolic bone disease, such as osteoporosis, we screened several concentrations of DM by performing tartrate-resistant acid phosphatase (TRAP) staining. Our study confirmed that a distilled water extract of DM significantly inhibits the formation of TRAP-positive osteoclasts. Therefore, we further investigated the pharmacological effects of DM on receptor activator of nuclear factor kappa-B ligand (RANKL)-induced osteoclast differentiation *in vitro* and its underlying molecular mechanisms. In addition, we investigated whether DM has restorative effects on lipopolysaccharide (LPS)-mediated bone loss *in vivo*.

## 2. Results and Discussion

### 2.1. DM Inhibits RANKL-Induced Osteoclastogenesis without Regulation of Early Signaling Pathways

**Figure 1:**
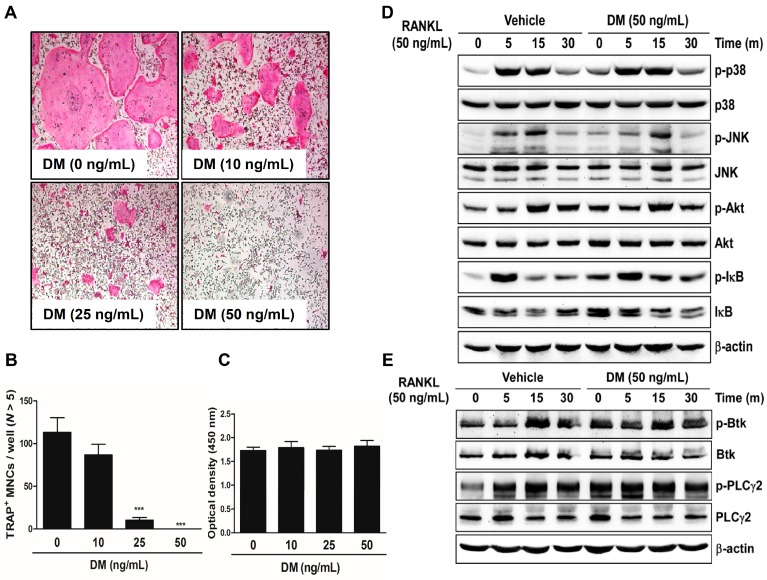
DM inhibits RANKL-induced osteoclast differentiation. (**A**) BMMs were cultured for 4 days with M-CSF (30 ng/mL) and RANKL (50 ng/mL) in the presence or absence of DM. Cells were fixed in 3.7% formalin, permeabilized with 0.1% Triton X-100, and stained with TRAP solution. TRAP-positive multinucleated cells (TRAP^+^ MNCs) were photographed under a light microscope; (**B**) TRAP^+^ MNCs with more than five nuclei were counted. *** *p* < 0.001 *vs.* control; (**C**) BMMs were seeded into a 96-well plate and cultured for 3 days in the presence of M-CSF (30 ng/mL) and with the indicated concentrations of DM. After 3 days, the absorbance was measured at 450 nm using an ELISA reader; (**D**,**E**) BMMs were cultured for 1 day in the presence of M-CSF (10 ng/mL). Afterwards, BMMs were starved for 3 h, pretreated with DM (50 ng/mL) for 1 h, and then stimulated with RANKL (50 ng/mL) for the indicated times. Cell lysates were analyzed by western blotting with antibody against p-p38, p38, p-JNK, JNK, p-Akt, Akt, p-IκB, IκB, p-Btk, Btk, p-PLCγ2, PLCγ2, and β-actin.

We cultured mouse-derived primary bone marrow macrophages (BMMs) in the presence of macrophage colony-stimulating factor (M-CSF) and RANKL with the indicated concentrations of DM to demonstrate that DM can influence the formation of TRAP-positive multinucleated cells (MNCs). DM suppressed RANKL-mediated osteoclast differentiation in a dose-dependent manner (Figure 1A). The number of TRAP-positive MNCs was significantly reduced after DM treatment (Figure 1B). This anti-osteoclastogenic effect was exerted without any cytotoxic effects (Figure 1C). In the early stages of osteoclast differentiation, several types of signal transduction pathways including the proteins p38, c-jun *N*-terminal kinase (JNK), Akt, and IκB are activated in response to the direct binding of RANK, a cell surface marker of osteoclast precursors, and RANKL, a crucial cytokine mediator of osteoclast development [11,12,13,14,15]. Ca^2+^ signaling is required for RANKL-dependent osteoclastogenesis via phosphorylation of Bruton’s tyrosine kinase (Btk) and, further downstream, phospholipase C (PLC)γ2 [16]. However, RANKL-induced phosphorylation of these signaling pathways was not associated with the anti-osteoclastogenic effect of DM (Figure 1D).

### 2.2. DM Down-Regulates Expression Levels of c-Fos and NFATc1, and Subsequently Reduced the Levels of Osteoclast-Specific Marker Genes

In response to the activation of a variety of RANKL-dependent transducers, such as mitogen-activated protein kinases (MAPKs), nuclear factor-kappa B (NF-κB), and phosphatidylinositol 3 (PI3)-kinase, induction of c-Fos and nuclear factor of activated T cells c1 (NFATc1), which are essential transcription factors in osteoclasts, is achieved [17]. c-Fos belongs to a family of dimeric transcription factors, including activator protein-1 (AP-1), and it has been proven to play an essential role in osteoclast-associated signal transduction, proliferation, and differentiation. Mice lacking c-Fos (generated from gene targeting in embryonic stem cells) exhibit abnormal bone phenotypes such as osteopetrosis, owing to insufficient osteoclast activity [18].

**Figure 2:**
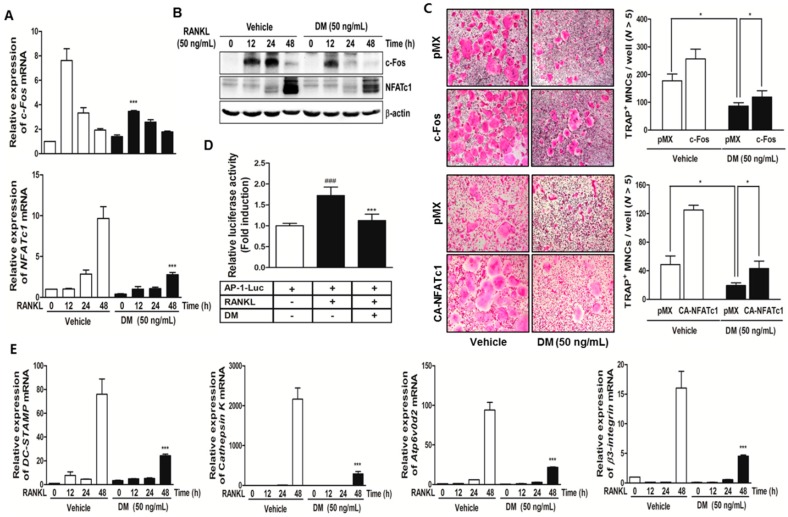
DM suppresses the expressions of c-Fos, NFATc1, and other osteoclast marker genes. (**A**) BMMs were stimulated with RANKL (50 ng/mL) and M-CSF (30 ng/mL) in the presence or absence of DM (50 ng/mL) for the indicated times. Total RNA was isolated from cells using QIAzol reagent; mRNA expression levels of c-Fos and NFATc1 were evaluated using quantitative real-time RT-PCR. *** *p* < 0.001 *vs.* control in the indicated time. *** *p* < 0.001 *vs.* control in the indicated time; (**B**) BMMs were pretreated with or without DM (50 ng/mL) for 1 h and then stimulated with M-CSF (30 ng/mL) and RANKL (50 ng/mL) for the indicated times. The cell lysates were analyzed by western blotting with c-Fos, NFATc1, and β-actin antibodies; (**C**) BMMs were infected with retroviruses expressing pMX-IRES-EGFP (pMX), pMX-CA-NFATc1-EGFP, and pMX-c-Fos-EGFP. Infected BMMs were cultured with or without DM (50 ng/mL) in the presence of M-CSF (30 ng/mL) and RANKL (50 ng/mL) for 4 days. After culturing, the cells were fixed and stained for TRAP (left). The TRAP^+^ MNCs with more than five nuclei were counted (right). * *p* < 0.05 *vs.* the indicated group; (**D**) HEK293T cells were cotransfected with RANK and AP-1 luciferase plasmid. At 12 h after transfection, the cells were pretreated with DM (50 ng/mL) or vehicle (DMSO) for 24 h and then stimulated with RANKL (100 ng/mL) for 24 h. The cells were lysed and the luciferase activity was assayed. ^###^ *p* < 0.001 *vs.* RANKL-untreated group and *** *p* < 0.001 *vs.* RANKL-treated group; (**E**) BMMs were stimulated with RANKL (50 ng/mL) and M-CSF (30 ng/mL) in the presence or absence of DM (50 ng/mL) for the indicated times. Total RNA was isolated from the cells using QIAzol reagent; mRNA expression levels of *Cathepsin K*, *β3-integrin*, *DC-STAMP*, and *Atp6vOd2* were evaluated using quantitative real-time RT-PCR. *** *p* < 0.001 *vs.* control at the indicated time.

During osteoclast differentiation, c-Fos induces a transcriptional regulatory cascade by cooperating with another transcription factor, NFATc1. Previous reports demonstrated that the transfer of an active form of NFATc1 in c-Fos-deficient osteoclast precursors leads to normal osteoclast formation and bone-resorbing activity [19]. In addition, NFATc1 positively controls the differentiation of monocytes and macrophages into mature osteoclasts, even in the absence of RANKL stimulation [17]. In this study, we confirmed that DM down-regulated the expressions of c-Fos and NFATc1 at both mRNA (Figure 2A) and protein levels (Figure 2B), and only ectopic expression of these two factors opposed the anti-osteoclastogenic action of DM (Figure 2C). Furthermore, we examined for direct evidence that DM inhibits the c-Fos transcriptional activity by cotransfecting HEK293T cells with RANK and the AP-1 promoter luciferase gene. RANK-mediated AP-1 promoter activity was inhibited in the presence of DM (Figure 2D). These results suggest that DM is involved in RANKL-mediated osteoclast differentiation via direct targeting of c-Fos and subsequent reduction of NFATc1 expressions. NFATc1 regulation by c-Fos during osteoclastogenesis is required for the expression of osteoclast-specific genes. As shown Figure 2E, the expression of several osteoclast-specific markers, including *cathepsin K*, *β3-integrin*, *dendritic cell-specific transmembrane protein* (*DC-STAMP*), and *Atp6v0d2* was markedly inhibited by DM treatment.

### 2.3. DM Deteriorates F-actin Ring Formation and Bone-Resorbing Activity of Mature Osteoclasts

**Figure 3:**
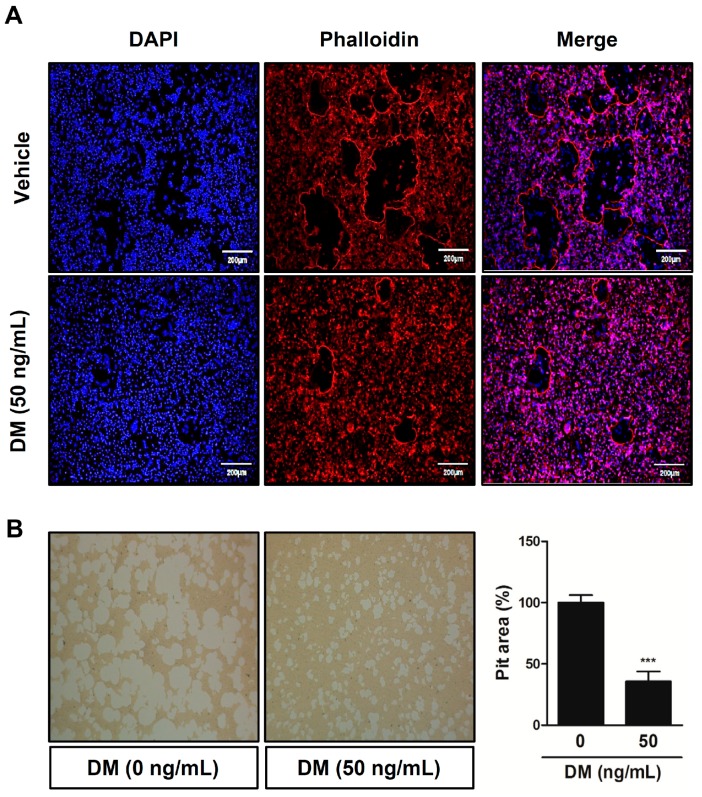
DM suppresses F-actin ring formation and bone-resorbing activity of mature osteoclasts. (**A**) BMMs were cultured for 4 days in the presence of M-CSF (30 ng/mL) and RANKL (50 ng/mL) with or without various concentrations of DM. Cells were fixed with 3.7% formalin, permeabilized with 0.1% Triton X-100, and stained with phalloidin (for the actin ring) and DAPI (for the nucleus). Images were obtained by confocal microscopy at 20× magnification; (**B**) Mature osteoclasts were seeded on hydroxyapatite-coated plates for 24 h with the indicated concentrations of DM. Attached cells on the plates were removed and photographed under a light microscope (20×). The relative ratio pit areas was quantified using Image J. *** *p* < 0.001 *vs.* control.

At the later stages of osteoclast maturation, changes in osteoclast morphology are induced by organizing cytoskeletal filamentous actin (F-actin) into a specific structure in order to adhere to the surface of the mineralized bone matrix. With the initiation of the formation of a belt-like F-actin ring (also called the sealing zone), the osteoclast can establish an acidic microenvironment between extracellular matrix and its ruffled border that has prominent subdomains for osteoclastic bone resorption. In the region between the osteoclast and the bone surface, the cell’s ruffled border activates a vacuolar H(+)-ATPase-type proton pump to secrete hydrogen ions and proteolytic enzymes, such as Cathepsin K, resulting in degradation of the bone matrix [20,21,22,23,24]. In this study, we observed that DM disrupted the formation of the F-actin ring-positive osteoclast, subsequently attenuating the bone-resorbing activity of mature osteoclasts (Figure 3).

### 2.4. Oral Administration of DM Exhibits a Restoration Effect on Bone Loss Induced by Intraperitoneal Injection with LPS in Vivo

**Figure 4:**
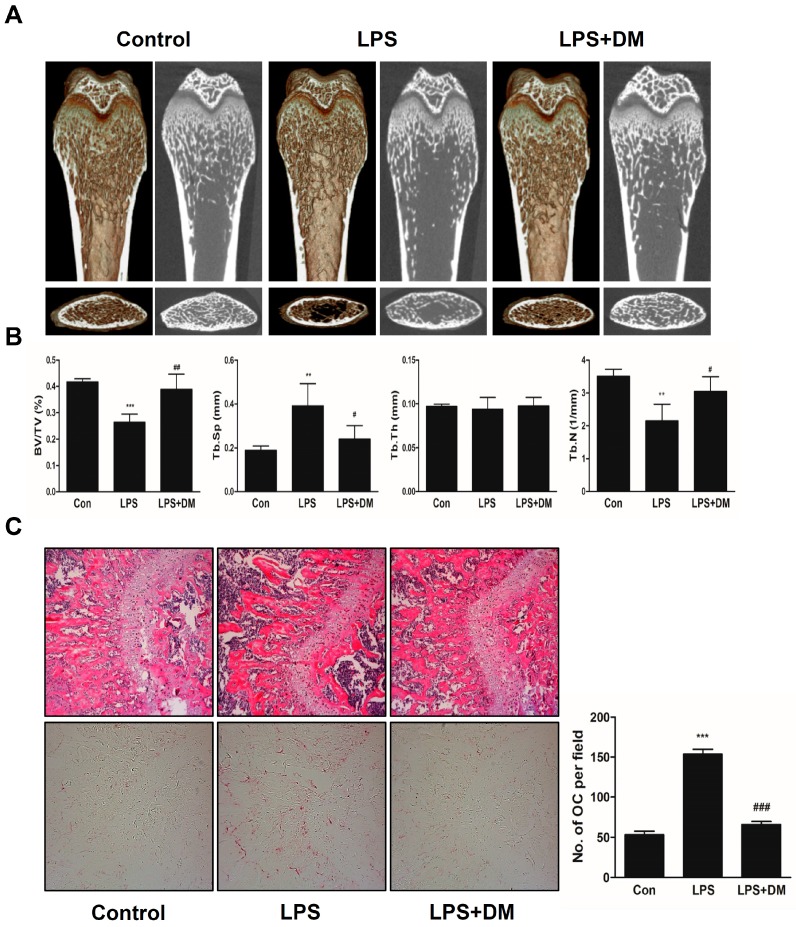
DM exhibits a restoration effect on LPS-mediated bone-loss mouse model. (**A**) Mice were sacrificed 8 days after the first LPS injection; 2D or 3D radiographs of the coronal and transverse planes of the proximal femora were obtained by μ-CT; (**B**) BV/TV, Tb.Sp, Tb.Th, and Tb.N of the femora were determined using the μ-CT data and analyzed using INFINITT-Xelis software. ** *p* < 0.01, *** *p* < 0.001 *vs.* control; ^#^ *p* < 0.05, ^##^ *p* < 0.01 *vs.* LPS group; (**C**) Dissected femora were fixed, decalcified, embedded, and sectioned. Sections were stained with TRAP and H & E. The number of osteoclasts per field of tissue was measured by performing histomorphometric analysis. *** *p* < 0.001 *vs.* control; ^###^ *p* < 0.01 *vs.* LPS group.

Based on the inhibitory effects of DM on osteoclast differentiation and function *in vitro*, we further performed an *in vivo* study. LPS is an important mediator of pathological bone destruction associated with inflammation. LPS leads to the intracellular activation of p38, JNK, and NFκB in macrophages and monocytes, and promotes the differentiation and survival of osteoclasts through the production of other factors such as PGE_2_, interleukin 1, RANKL, and TNF [25]. Among the several kinds of osteoporosis mice model, we employed the inflammation-induced bone loss model through injection of LPS on the grounds that DM had anti-inflammatory effect [7]. Mice were intraperitoneally injected with PBS as a control, and LPS to produce a bone loss model. After 8 days, the left femora of the sacrificed mice were used to acquire three-dimensional (3D) images through micro-computed tomography (μ-CT) analysis. As shown in Figure 4A, a considerable reduction in bone mass was observed in the femora of LPS-treated mice, in comparison with the PBS-treated control mice. However, the loss of trabecular bone was partially restored in LPS- and DM-treated mice. Morphometric analysis of the femora of LPS-injected mice exhibited a decrease in bone volume per tissue volume (BV/TV) and trabecular number (Tb.N), and an increase in trabecular separation (Tb.Sp). However, these changes were significantly attenuated in LPS- and DM-treated mice (Figure 4B). Additionally, DM treatment suppressed LPS-induced degradation of bone matrix and TRAP-positive osteoclast formation within regions of the growth plate (Figure 4C).

## 3. Materials and Methods

### 3.1. Reagents and Antibodies

The distilled water extract of DM was purchased from the Korean Plant Extract Bank (Daejeon, Korea). The TRAP staining solution was purchased from Sigma Aldrich (St. Louis, MO, USA), and the XTT assay kit was obtained from Roche (Indianapolis, IN, USA). The α-minimum essential medium (α-MEM), fetal bovine serum (FBS), and penicillin-streptomycin were purchased from Gibco-BRL (Grand Island, NY, USA); soluble human recombinant M-CSF and RANKL were purchased from Peprotech (London, UK). Specific primary antibodies against phospho-p38 (#9211), p38 (#9212), phospho-JNK (#9251), JNK (#9252), phospho-Akt (#9271), Akt (#9272), and phospho-IκB (#2859) were purchased from Cell Signaling Technology (Beverly, MA, USA), while phosphor-Btk (GTX61791) was purchased from GeneTex (Irvine, CA, USA) and β-actin (A5441; housekeeping gene) was obtained from Sigma Aldrich. Specific antibodies against phospho-PLCγ2 (sc-101785), PLCγ2 (sc-5283), IκB (sc-371), and c-Fos (sc-7202), and NFATc1 (sc-7294) were obtained from Santa Cruz Biotechnology (Santa Cruz, CA, USA).

### 3.2. Mouse Bone Marrow Cells Isolation and Osteoclast Differentiation

Mouse bone marrow cells (BMCs) were obtained from the femora and tibiae of 5-week-old ICR mice and incubated in α-MEM with 10% FBS, 1% penicillin/streptomycin, and M-CSF (10 ng/mL) for 1 day to obtain non-adherent cells. The non-adherent cells were incubated in α-MEM with 10% FBS, 1% penicillin/streptomycin, and M-CSF (30 ng/mL) as osteoclast precursors for 3 days. After 3 days, the adherent cells were used as BMMs. The BMMs were incubated with M-CSF (30 ng/mL) and RANKL (50 ng/mL) in the presence of DM (0–50 ng/mL). After 3 days, the culture media was changed under the same conditions. After 1 day, the cells were fixed in 3.7% formalin for 20 min, permeabilized with 0.1% Triton X-100, and then stained with TRAP solution. The stained cells were counted to establish the level of osteoclast differentiation.

### 3.3. Cell Viability Assay

The BMMs (1 × 10^4^ cells/well) were cultured with or without DM (10–50 ng/mL) for 3 days in the presence of M-CSF (30 ng/mL) in 96-well plates. After 4 h of incubation in a medium containing 50 μL of XTT solution (sodium 3′-[1-(phenylaminocarbonyl)-3,4-tetrazolium]-bis(4-methoxy-6-nitro), benzenesulfonic acid hydrate, and *N*-methyldibenzopyrazine methyl sulfate), the optical density of the cells was measured at 450 nm using an enzyme-linked immunosorbent assay (ELISA) reader (Molecular Devices, Sunnyvale, CA, USA).

### 3.4. Western Blot Analysis

The BMMs were lysed in a lysis buffer containing 50 mM Tris-HCl, 150 mM NaCl, 5 mM ethylenediaminetetraacetic acid (EDTA), 1% Triton X-100, 1 mM sodium fluoride, 1 mM sodium vanadate, 1% deoxycholate, and protease inhibitors. The lysate was centrifuged at 14,000 rpm for 20 min to obtain pure protein. The protein concentration was measured using a Bio-Rad colorimetric protein assay kit (Bio-Rad Laboratories Inc., Hercules, CA, USA), and equal amounts of protein were separated with an SDS-polyacrylamide gel. The proteins were transferred to a polyvinylidene difluoride (PVDF) membrane (Millipore, Bedford, MA, USA) and treated with 5% non-fat dry milk to inhibit the attachment of non-specific proteins. After the membrane was treated with primary and secondary antibodies (horseradish peroxidase [HRP]-conjugated sheep anti-mouse or donkey anti-rabbit immunoglobulin), the expression of specific protein signals was measured using a chemiluminescence detection system (Millipore).

### 3.5. Quantitative Real-Time RT-PCR Analysis

Total RNA was extracted using the QIAzol lysis reagent (Qiagen, Valencia, CA, USA) according to the manufacturer’s instructions. Equal amounts of cDNA were synthesized from 1 μg of total RNA using SuperScript II Reverse Transcriptase (Invitrogen, San Diego, CA, USA). Real-time RT-PCR was performed using an Exicycler™ 96 Real-Time Quantitative Thermal Block (Bioneer Co., Daejeon, Korea) with a 20-μL reaction mixture containing 10 μL SYBR Green Premix (Bioneer Co.), 10 pmol forward primer, 10 pmol reverse primer, and 1 μg cDNA. The real-time RT-PCR detection program proceeded according to the following protocols: initial denaturation at 95 °C for 5 min, and 40 cycles of 3-step PCR (denaturation at 95 °C for 1 min, annealing at 60 °C for 30 s, and extension at 72 °C for 1 min). Gene expression levels were normalized to the housekeeping gene, β-actin. The relative results of specific genes were calculated using the comparative cycle threshold method. Table 1 shows the primer sets used in the real-time RT-PCR.

### 3.6. Retroviral Gene Transfection

Packaging of the retroviral vectors pMX-IRES-EGFP, pMX-cFos-IRES-EGFP, and pMX-constitutively active (CA)-NFATc1-IRES-EGFP was performed via transient transfection of these pMX vectors into Plat-E retroviral packaging cells using X-tremeGENE 9 (Roche, Nutley, NJ, USA), according to the manufacturer’s protocol. After incubation in fresh medium for 2 days, the culture supernatants of the retrovirus-producing cells were collected. For retroviral infection, non-adherent BMCs were cultured in M-CSF (30 ng/mL) for 2 days. The BMMs were incubated with viral supernatant pMX-IRES-EGFP, pMX-cFos-IRES-EGFP, and pMX-CA-NFATc1-IRES-EGFP virus-producing Plat-E cells together with polybrene (10 μg/mL) and M-CSF (30 ng/mL) for 6 h. The infection efficiency of the retrovirus was determined by green fluorescent protein expression, and it was found to be >80%. After infection, the BMMs were induced to differentiate in the presence of M-CSF (30 ng/mL) and RANKL (50 ng/mL) for 4 days. The forced expression of each construct and the resulting osteoclast formation were detected using a fluorescence microscope and TRAP staining.

### 3.7. Luciferase Reporter Assay

For transfection of reporter plasmids, HEK293T cells were plated in 24-well plate (4 × 10^4^ cells/well) and incubated for 24 h prior to transfection. Cells reporter were cotransfected with RANK and AP-1 luciferase reporter vector by X-tremeGENE 9 for 6 h in serum-free DMEM, and then the medium was replaced by DMEM complete medium. At 12 h after transfection, the cells were pretreated with DM (50 ng/mL) or vehicle (DMSO) for 24 h and then stimulated with RANKL (100 ng/mL) for 24 h. The cells were lysed with lysis buffer (Promega, Madison, WI, USA) and luciferase activity was measured using a luciferase assay system (Promega) according to the manufacturer’s instructions. Luciferase activity was normalized to the β-galactosidase activity in each sample.

### 3.8. Actin Ring Staining

BMMs were cultured for 3 days with M-CSF (30 ng/mL) and RANKL (50 ng/mL) in the presence or absence of DM (50 ng/mL). Cells were fixed with PBS containing 3.7% formaldehyde and permeabilized with PBS containing 0.1% Triton X-100. The cells were blocked using 2.5% bovine serum albumin (BSA), incubated with phalloidin (Molecular Probes, Eugene, OR, USA) at room temperature for 30 min, and washed and rinsed with PBS before mounting with 4′,6-Diamidino-2-phenyl-indole (DAPI) (Sigma, Steinheim, Germany). Images were taken using a fluorescence microscope (DMLB, Leica, Wetzlar, Germany).

### 3.9. Bone Resorption Assay

To obtain mature osteoclasts, BMCs and primary osteoblasts were co-cultured in collagen gel-coated culture dishes for 7 days in the presence of 1,25-dihydroxyvitamin D_3_ (VitD_3_; Sigma) and prostaglandin E2 (PGE2; Sigma). After 7 days, the co-cultured cells were detached using 0.1% collagenase at 37 °C for 10 min, and the separated cells were replated on hydroxyapatite-coated plates (Corning, New York, NY, USA) with or without DM (50 ng/mL) for 24 h. Afterwards, the cells on the plates were removed and the resorption pits were photographed and quantified using the Image-Pro Plus program version 4.0 (Media Cybernetics, Silver Spring, MD, USA).

### 3.10. Mouse Model of LPS-Induced Bone Erosion and μ-CT and Histological Analysis

Five-week-old male ICR mice were purchased from Samtako Inc. (Osan, Korea). The mice were kept in a temperature-(22–24 °C) and humidity-(55%–60%) controlled environment with a 12 h light/dark cycle. The use of experimental animals was reviewed by the IACUC and approved under WKU15-92. The ICR mice were divided into 3 main experimental groups with 5 mice each: PBS-treated (Control), only LPS-treated (LPS), and both LPS and DM-treated (LPS+DM). DM is administrated at the dosage of 200 mg/kg body weight in reference to previous report [8]. DM (200 mg/kg) or PBS was administered orally 1 day before LPS injection (5 mg/kg). DM or PBS was administered orally every 8 days and there were no unusually clinical indications for a period of DM administration. To induce acute inflammation response, LPS was injected intraperitoneally on days 1 and 4. The mice were sacrificed after 8 days and their left femora underwent high-resolution μ-CT analysis. The intact femur metaphysic regions were scanned by μ-CT (NFR-Polaris-S160; Nanofocus Ray, Iksan, Korea) with a source voltage of 60 kVp, current of 114 mA, and 7 mm isotropic resolution. Femur scans were performed from the growth plate to approximately 2 mm, with a total of 350 sections per scan. After 3D reconstruction, BV/TV, Tb.N, Tb.Th, and Tb.Sp were calculated using INFINITT-Xelis software (INFINITT Healthcare, Seoul, Korea). The right femora were fixed in 4% neutral buffered paraformaldehyde (Sigma) for 1 day, decalcified for 3 weeks in 12% EDTA, and embedded in paraffin. Sections of 5-μm thickness were prepared using a Leica microtome RM2255 (Leica Microsystems, Bannockburn, IL, USA). For histologic examination, sections were stained with hematoxylin and eosin (H & E); another section was stained with TRAP to identify osteoclasts on the bone surface. Parameters for bone resorption, including number of osteoclasts per field of tissue, were quantified using the Image Pro-Plus program, version 4.0 (Media Cybernetics, Silver Spring, MD, USA). Nomenclature, symbols, and units used in this study are those recommended by the American Society for Bone Mineral Research (ASBMR) Nomenclature Committee.

### 3.11. Statistical Analysis

All experiments were conducted in at least triplicate, and the data are expressed as mean ± standard deviation (SD). All statistical analyses were performed using Statistical Package for the Social Sciences Software (SPSS; Korean version 14.0). Student’s *t*-test was used to compare the parameters between two groups, while analysis of variance followed by Tukey’s *post-hoc* test was used to compare parameters among 3 groups. *p* < 0.05 was considered statistically significant.

## 4. Conclusions

DM attenuated RANKL-induced osteoclast differentiation in a dose-dependent manner without any cytotoxic effects via direct down-regulation of the expression levels of c-Fos and NFATc1. This anti-osteoclastogenic action interrupted the formation of the F-actin structure, which is a prerequisite for the development of functional osteoclasts, and caused impaired bone-resorbing activity by mature osteoclasts. Reflecting these *in vitro* effects, administration of DM attenuated LPS-mediated inflammatory bone erosion *in vivo*. Taken together, the present study is the first to suggest that DM has potential in the treatment of bone metabolic diseases such as osteoporosis.

## Figures and Tables

**Table 1:** Primer sequences used for real-time RT-PCR analysis.

Gene Name		Primer Sequence (5′ → 3′)
*GAPDH*	Forward	5′-TCA AGA AGG TGG TGA AGC AG-3′
	Reverse	5′-AGT GGG AGT TGC TGT TGA AGT-3′
*c-Fos*	Forward	5′-GGT GAA GAC CGT GTC AGG AG-3′
	Reverse	5′-TAT TCC GTT CCC TTC GGA TT-3′
*NFATc1*	Forward	5′-GAG TAC ACC TTC CAG CAC CTT-3′
	Reverse	5′-TAT GAT GTC GGG GAA AGA GA-3′
*Cathepsin K*	Forward	5′-CCA GTG GGA GCT ATG GAA GA-3′
	Reverse	5′-CTC CAG GTT ATG GGC AGA GA-3′
*β* *3-integrin*	Forward	5′-GGA GTG GCT GAT CCA GAT GT-3′
	Reverse	5′-TCT GAC CAT CTT CCC TGT CC-3′
*Atp6vOd2*	Forward	5ʹ-GAC CCT GTG GCA CTT TTT GT-3ʹ
	Reverse	5ʹ-GTG TTT GAG CTT GGG GAG AA-3ʹ
*DC-STAMP*	Forward	5′-TCC TCC ATG AAC AAA CAG TTC CA-3′
	Reverse	5′-AGA CGT GGT TTA GGA ATG CAG CTC-3′
